# 

**Published:** 1887-03-19

**Authors:** 


					\w ? PRICE ONE PENNY. n
\
No. 25, Vol. I.
March 19th, 1887.
s'siereu at tlie Oeneral Post Office
as a Newspaper.]
mm
Per Annum, 6s. 6d.,post free.
Monthly Parts, 6d.; post free, 7id.
Ditto per Ann., 7s. 6d., post free.
znd farKicXJfasMlgl
an Enstttution, .ffamilg, attlr $arocf)taI journal
hospitals, &s?Iums, & all agencies for tfje (Hare of tfje Sicfc, Criticism & jletos.

				

